# Supramolecular Detection of a Nerve Agent Simulant by Fluorescent Zn–Salen Oligomer Receptors

**DOI:** 10.3390/molecules24112160

**Published:** 2019-06-08

**Authors:** Roberta Puglisi, Placido G. Mineo, Andrea Pappalardo, Antonino Gulino, Giuseppe Trusso Sfrazzetto

**Affiliations:** 1 Department of Chemical Sciences, University of Catania, Viale A. Doria 6, 95100 Catania, Italy; gmineo@unict.it (P.G.M.); andrea.pappalardo@unict.it (A.P.); agulino@unict.it (A.G.); 2 CNR-IPCB Istituto per i Polimeri, Compositi e Biomateriali, Via P. Gaifami 18, I-95126 Catania, Italy; 3 Department of Chemical Sciences, University of Catania Research Unit (I.N.S.T.M.) UdR of Catania, Viale A. Doria 6, 95125 Catania, Italy

**Keywords:** chemical warfare agents, supramolecular detection, oligo-Salen

## Abstract

We report on new Zn–Salen oligomer receptors able to recognize a nerve agent simulant, namely dimethyl methylphosphonate (DMMP), by a supramolecular approach. In particular, three Zn-Salen oligomers (Zn–Oligo–A, –B, and –C), differing by the length distribution, were obtained and characterized by NMR, Gel Permeation Chromatography (GPC), UV-Vis, and fluorescence spectroscopy. Furthermore, we investigated their recognition properties towards DMMP by using fluorescence measurements. We found that the recognition ability depends on the length of the oligomeric chain, and the Zn–Oligo–C shows a binding constant value higher than those already reported in literature for the DMMP detection.

## 1. Introduction

**Figure 1:**
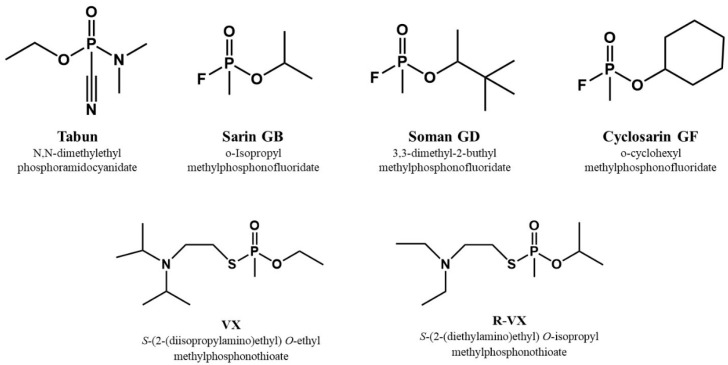
Nerve agents of G (top) and V (bottom) series.

The nerve agents (NAs) of G and V series (Figure 1) are the most toxic known organophosphorus (OP) derivatives, synthesized for the first time as pesticides and then used as chemical weapons during world conflicts or terroristic attacks. They are strong acetylcholinesterase inhibitors and cause cholinergic crisis in the human body, after breath exposure [1,2].

G-serie NAs are highly volatile (for example, Tabun, Sarin, and Soman show a volatility in the 576–22,000 mg/m^3^ range at 25 °C), while the V-serie shows lower volatility (3–30 mg/m^3^ at 25 °C). For these reasons, NA can be released in air or in water. These compounds start to be toxic in the 25–400 mg × min/m^3^ range. For these reasons, a good sensor should be able to detect NA both in solution and in gas phase, with a detection limit of few ppm.

Although use, synthesis, and storage of chemical warfare agents (CWAs) are forbidden for security reasons, OP compounds, such as Sarin, Soman, and the more recent Novichock [3] are still used as weapons to injure or kill people, as demonstrated by some recent international events [4,5].

With the aim to increase human health and safety, many efforts have been made to develop new and more efficient detection systems, able to reveal the presence of OP compounds in low concentrations and to give a measurable and observable response of NA presence [6].

**Figure 2:**
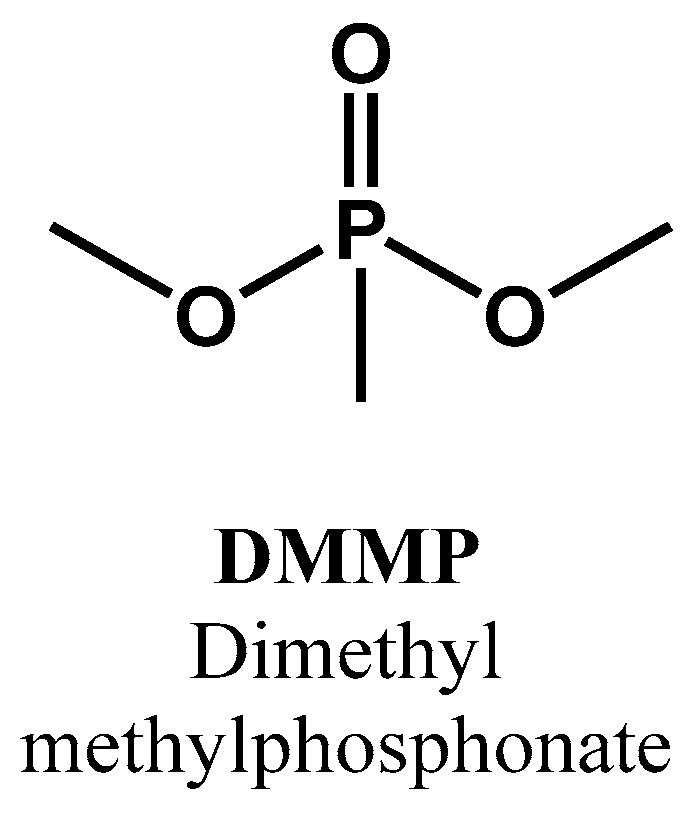
DMMP structure.

Due to their high toxicity, the use of nerve agents is not even allowed for research activity. For this reason, studies on new molecular receptors are carried out using simulants—less toxic organophosphorus compounds. In this context, recent studies demonstrated that dimethyl methylphosphonate (DMMP, Figure 2) is one of the best simulants for “G” series nerve agents [7].

Instrumental recognition of nerve gases (such as mass spectrometry or gas chromatography) is highly sensitive [8,9]. However, the complexity of the systems involved is a limit for a “real field” application. In this context, molecular probes represent a more convenient detection strategy [10]. Today, molecular sensing of chemical warfare agents can be performed by two main different methods: (*i*) A covalent approach, in which the receptor covalently binds the analyte, thus giving a measurable response (such as a change of the fluorescence emission) after the interaction [11,12]. Due to the nature of the interactions involved, covalent receptors are not reusable after a first exposure to nerve agents, and false positive responses can be obtained; (*ii*) the more recent and less studied supramolecular approach to detect NA by non-covalent reversible interactions, thus leading to a supramolecular reversible host–guest complex and reusable sensors [13].

To date, only a few examples of supramolecular hosts for CWA simulants have been reported in the literature [14,15,16,17,18,19,20,21,22,23,24]. They exploit several non-covalent interactions for the recognition of the guest, such as inclusion within the hydrophobic macrocyclic cavity [14,15,16,17] or hydrogen bond-dependent gel formation [18,19,20,21,22,23,24,25].

Metal–Salen complexes are well known in literature for their use as catalysts [26,27,28,29,30] and supramolecular hosts [31,32,33,34,35,36,37,38], where the Lewis acid metal center is exploited for the recognition of Lewis base species. Furthermore, recently we also demonstrated the high efficiency of metal-Salen complexes as receptors for NA simulants [25,39]. In particular, we reported on uranyl–Salen complexes able to efficiently recognize DMMP with high affinity constants due to a Lewis acid–base interaction between the metal center and the phosphate group [25]. In order to obtain a fluorescence response, we explored the use of Zn–Salen complexes for the DMMP detection, and achieved excellent results in terms of affinity and selectivity [39].

Encouraged by these results, in this work we investigated both the multivalence effect [40], derived from the use of a large number of available recognition sites linked to each other in Zn-Salen chains, and the additivity effect of the individual binding contribution in the detection of DMMP. To the best of our knowledge, this is the first example of oligomer-based Salen able to detect CWA simulants in solution.

**Figure 3:**
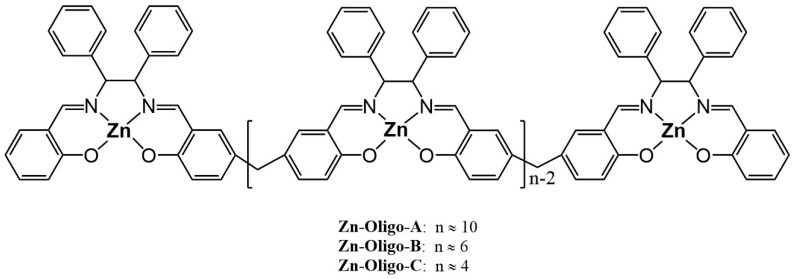
Schematic representation of the Zn–Salen oligomer hosts.

To this aim, we reported here the synthesis, characterization, and DMMP recognition abilities of three different Zn–Salen oligomers (Zn–Oligo–A, –B, and –C, Figure 3), differing by the length of the oligomeric chain.

## 2. Results and Discussion

**Scheme 1:**
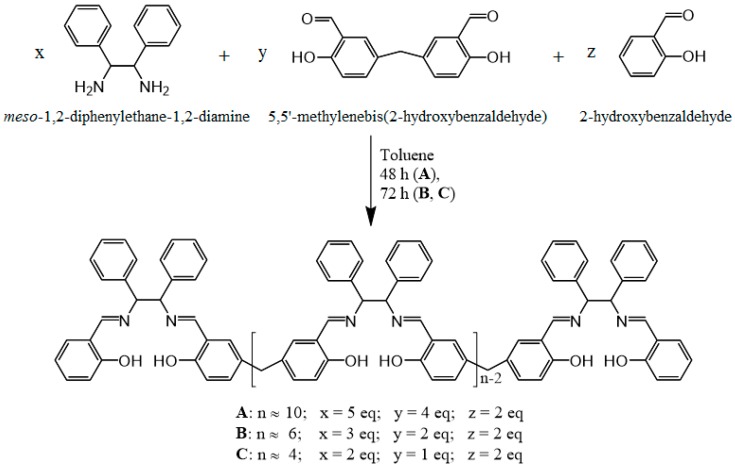
Schematic reaction pathways of the Oligo–Salen ligands.

Oligo–Salen ligands A, B, and C were easily synthesized in a one pot reaction, by mixing the proper ratios of (1,2–diphenyl)–ethylenediamine (x), 5,5′–methylenebis(2–hydroxybenzaldehyde) (y) [41], and 2–hydroxybenzaldehyde in toluene (z) (Scheme 1). The role of 2–hydroxybenzaldehyde in toluene is crucial to tune the length of the oligomeric chain.

**Figure 4:**
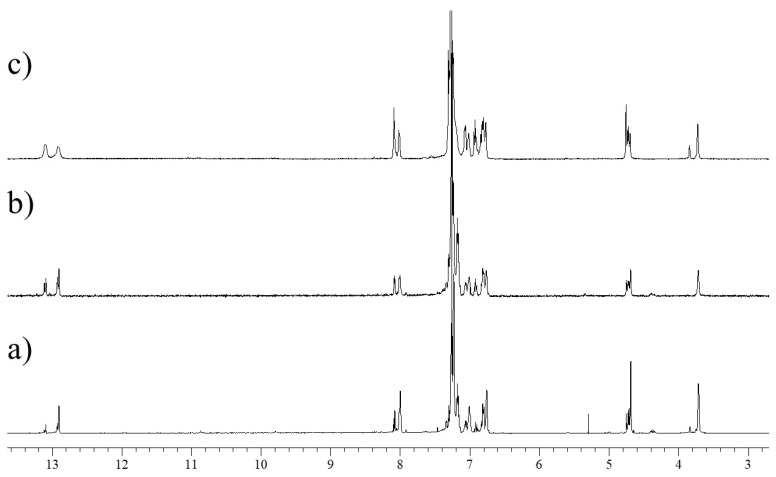
^1^H NMR spectra in CDCl_3_ of: (**a**) Oligo–Salen–A; (**b**) Oligo–Salen–B; (**c**) Oligo–Salen–C.

The reactions were monitored by TLC (Thin Layer Chromatography), following the disappearance of the starting reagent. The total conversions were reached after 48 h for A, 72 h for B and C. The oligo–Salen ligands A, B, and C were isolated by evaporation of the solvent and treatment with *n*-hexane. The isolated compounds showed similar ^1^H NMR spectra (Figure 4).

**Figure 5:**
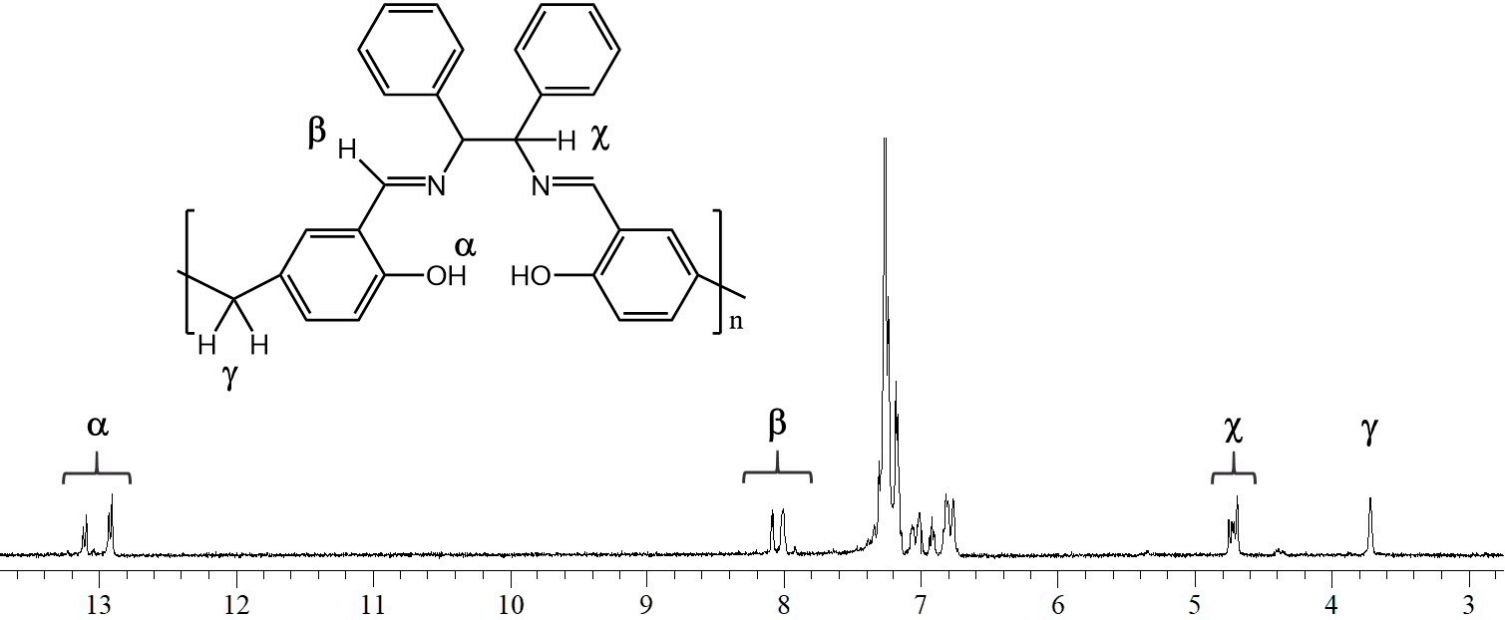
Representative ^1^H NMR spectrum of the Oligo–Salen–B (reported in the inset) in CDCl_3._

In particular, the following characteristic pattern of signals suggested the formation of the typical Salen backbone (Figure 5): (*i*) The disappearance of the aldehydic signal at ca. 10 ppm, (*ii*) the presence of signals at 8 ppm relative to the imine proton (β in Figure 5), and (*iii*) the downfield shift of hydroxyl protons signals, from 10.9 [41] to 13 ppm (α in Figure 5). In addition, the presence of methine (χ in Figure 5) and methylene protons (γ in Figure 5) at 4.8 and 3.7 ppm, respectively, confirmed the presence of the oligomeric chains.

Diffusion order spectroscopy is a useful NMR technique that can provide information about dimensions of analyzed species, measuring the diffusion coefficient [42,43]. DOSY measurements conducted in CDCl_3_ showed a diffusion coefficient of 3.41 × 10^−10^ m^2^s^−1^ for the Oligo–Salen–A (1 × 10^−3^ M) corresponding to an estimated molecular mass of 4650 Da, thus suggesting the prevailing presence of an oligomer containing ten units. Similarly, both Oligo–Salen–B and Oligo–Salen–C were analyzed by DOSY experiments in the same experimental conditions, and showed diffusion coefficients of 4.23 × 10^−10^ m^2^s^−1^ and 5.29 × 10^−10^ m^2^s^−1^, respectively. These data suggest the prevalent formation (in the oligomer mixtures) of a hexameric species for the Oligo–Salen–B and a tetrameric species for the Oligo–Salen–C (see Table 1).

In addition, ^1^H NMR spectra showed two patterns of signals relative to imine and OH groups: The downfield signals were related to the terminal aldehydic moiety of the oligomeric chain, while the upfield signals were relative to the inner core of the chain. The relative integration of these signals supported the identification performed by DOSY and Gel Permeation Chromatography GPC measurements.

To confirm the oligomeric nature of the obtained products and the decreasing of the averaging molecular weight by varying the reagents’ molar ratio, several GPC experiments were performed.

**Figure 6:**
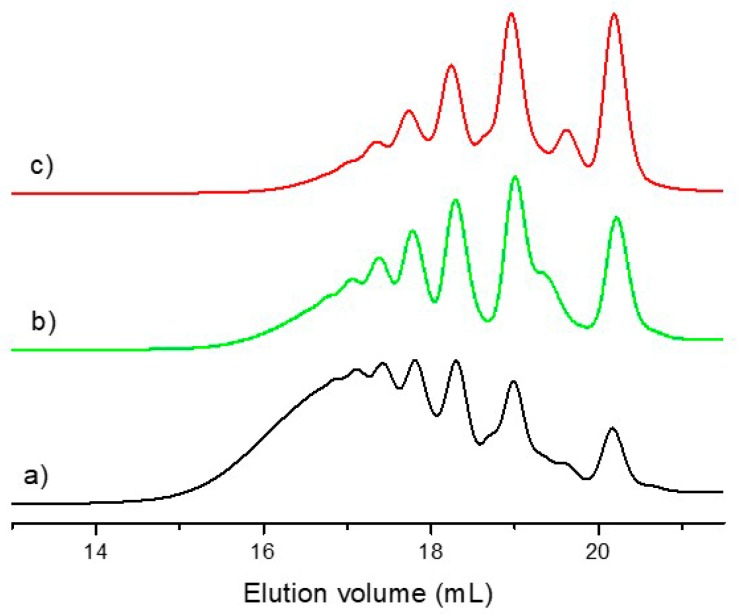
GPC traces of the Oligo–Salen–A, –B and –C compounds: (**a**) Oligo–Salen–A; (**b**) Oligo–Salen–B; (**c**) Oligo–Salen–C.

In particular, the GPC traces of the Oligo–Salen–A, –B and –C oligomeric mixtures (Figure 6) showed partially resolved peaks in the elution volume range 14.5–21.5 mL, certainly due to the presence of oligomeric species. It can be noticed that upon decreasing the amine/aldehyde molar ratio, the mass distribution curve moves toward higher elution volumes with, as expected, a quantitative enrichment of the oligomers at lower molar mass.

The oligomeric structures of the products were also confirmed by means of MALDI-TOF MS (see for example the spectrum of the Oligo–Salen–C reported in Figure S18 in the Supplementary Materials). This spectrum mainly consists of a series of peaks at *m*/*z* 851 + n432 (*n* = 0–7), detected as protonated species ([MH]^+^), that can be assigned to the molecular species reported in the inset of Figure 5.

**Scheme 2:**
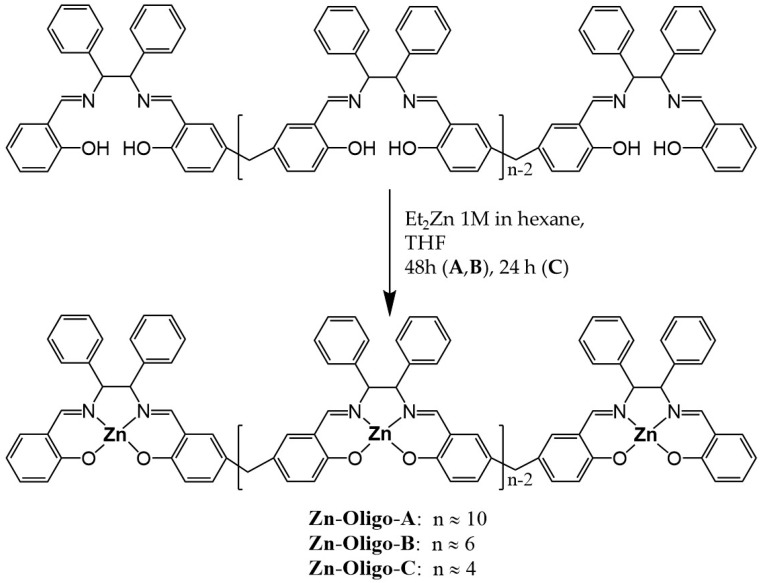
Synthesis of Zn–Salen oligomer hosts.

Metalation reactions of the three different Oligo–Salen compounds were performed in THF, by adding Zinc–Diethyl 1 M in hexane (Scheme 2) and collecting the final Zinc–Salen–oligomer complexes by filtration. ^1^H NMR spectra recorded in DMSO–*d*_6_ of Zn–Oligo–A, –B, and –C were similar. In particular, they showed a common upfield shift of the imine proton signals and the absence of hydroxyl group signals, thus supporting the formation of metal complexes.

**Figure 7:**
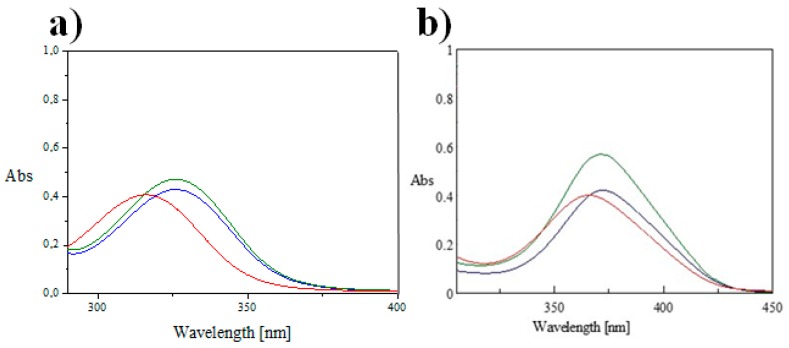
(**a**) UV–Vis spectra of Oligo–Salen–A (red), Oligo–Salen–B (blue), and Oligo–Salen–C (green) in DMSO (4 × 10^−5^ M); (**b**) Zn–Oligo–A (red), Zn–Oligo–B (blue), and Zn–Oligo–C (green) in DMSO (4 × 10^−5^ M).

UV–Vis absorbance spectra of oligomers showed the azomethine transition bands at ca. 325 nm (Figure 7a). Additionally, the corresponding Zn complexes showed similar profiles, with one main band consistent with the n–π* transitions (Figure 7b). In particular, the spectrum of Zn–Oligo–A recorded in DMSO showed a broad band at 365.2 nm (ε = 10,000 cm^−1^ M^−1^). Similarly, in the absorption spectrum of Zn–Oligo–B in DMSO, this band appeared at 372.0 nm (ε = 10,050 cm^−1^ M^−1^), while in the UV–Vis spectrum of Zn–Oligo–C it lay at 370.0 nm (ε = 14,600 cm^−1^ M^−1^).

**Figure 8:**
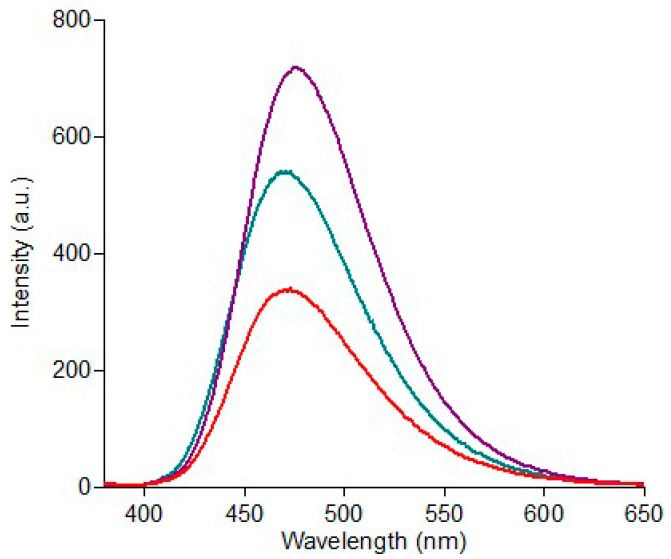
Emission spectra of Zn–Oligo–A (red), Zn–Oligo–B (green), and Zn–Oligo–C (purple) in DMSO (1 × 10^−5^ M, λ_ex_ = 365 nm).

Emission properties of Zn–oligomers were studied in DMSO. Using an excitation wavelength of 365 nm, similar fluorescence spectra were observed for all the three Zn–oligomers, with an intense emission band centered at ca. 480 nm (see Figure 8). The emission quantum yields calculated for the oligomers (φ = 0.31, 0.47, and 0.64 for Zn–Oligo–A, –B, and –C, respectively) showed a clear dependence on the length of the chains. The large Stokes shift (more than 100 nm) observed for these receptors paves the way for their sensing application.

**Figure 9:**
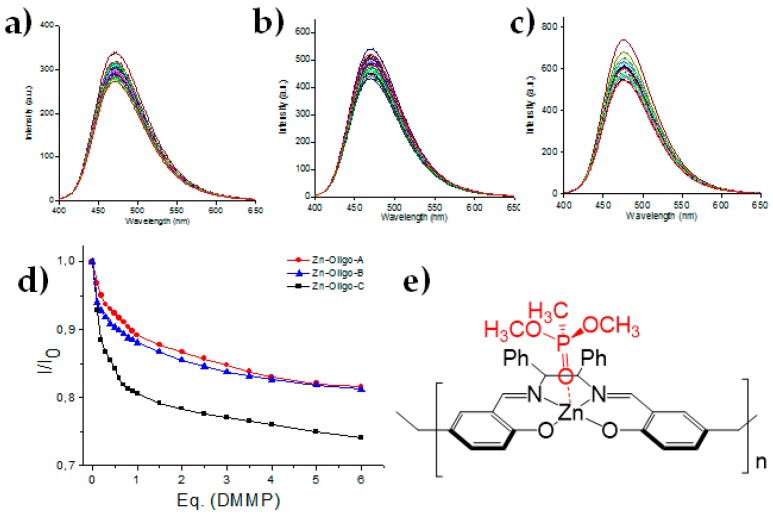
Fluorescence emission spectra of (**a**) Zn–Oligo–A, (**b**) Zn–Oligo–B, and (**c**) Zn–Oligo–C upon progressive addition of 0–6 eq. of DMMP ([receptors] = 1 × 10^−5^ M in DMSO, λ_ex_ 365 nm); (**d**) normalized fluorescence emission variation relative to Zn–Oligo–A (red), –B (blue), and –C (black) solutions (1 × 10^−5^ M in DMSO, λ_ex_ 365 nm), upon addition of DMMP (0–6 eq.); (**e**) supramolecular host–guest complex proposed.

Recognition studies of the three receptors towards DMMP were conducted by following the progressive quenching of the emission intensity, probably due to a PET (Photoinduced Electron Transder) mechanism [44], upon the addition of DMMP to a solution of each receptor (Figure 9a–c). As reported in Figure 9d, Zn–Oligo-C showed the highest emission quenching after the addition of DMMP. The limit of detection was 1 μM with all the receptors. The sensibility of Zn-Oligo hosts for DMMP is shown in Figure 9d, which highlighted the possibility to detect ppm (part per million) of DMMP. In particular, 1 ppm of DMMP produced an intensity emission variation of 25%–30%, if normalized with respect to the total observed variation, in Zn–Oligo–A and –C. This variation reached more than 70% with 10 ppm in the case of Zn–Oligo–C. Figure 9e shows the supramolecular complex proposed between Zn–oligomers and DMMP, supported by our previous studies in which 2D-NMR measurements suggested supramolecular geometry [39].

The data treatment using the HypSpec software provided the binding constant values for each receptor. Non-linear curve fit plots were performed by using a 1:1 stoichiometry, supported by our previous studies on CWA recognition by Metal–Salen complexes [31,32,33,34,35,36,37]. Interestingly, moving from longer to shorter oligomer distribution, an increase of the affinity was observed (see Table 2). In particular, we found that Zn–Oligo–C showed the highest affinity constant value towards DMMP and this value was larger than those already reported in the literature, even if compared with the monomeric form previously studied [39], due to the multivalence effect. However, the higher binding constant value of Zn–Oligo–C with respect to the longer receptors could be ascribed to energetic contributions. In particular, due to the presence in the oligomeric chain of the methylene groups, the shorter host could be more preorganized for the host–guest complex formation with respect to the longer receptors, which contains more methylene bridges.

Moreover, selectivity and competition tests were performed for the Zn–Oligo–C. In particular, atmospheric air (containing 24,000 ppm of water, 400 ppm CO_2_, 5 ppm NO, and 10 ppm CO) was bubbled for 10 min into a 1 × 10^−5^ M DMSO solution of Zn–Oligo–C. The emission spectra of the host were acquired before and after air bubbling, thus finding that the emission profile did not change after air exposure (see the Supplementary Materials). This experiment demonstrated that the emission properties of Zn–Oligo–C was not affected by the analytes/contaminants contained in the air. Then, this air-saturated solution was exposed to DMMP and we observed a decrease of the emission, thus confirming the ability of Zn–Oligo–C to also recognize DMMP in competitive conditions (see the Supplementary Materials).

**Figure 10:**
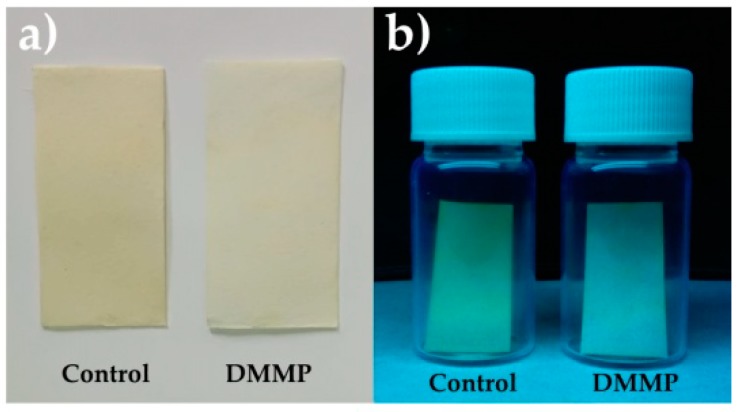
Test strip on filter paper (2.5 × 3 cm) adsorbed with the Zn–Oligo–C solution (1 × 10^−3^ M in DMSO), before (Control) and after (DMMP) the exposure to DMMP vapors (a 20 mL vial containing 1 μL of DMMP 0.1 M in CH_2_Cl_2_): (**a**) Under daylight; (**b**) under UV lamp irradiation (365 nm)**.**

In order to evaluate the use of Zn–Oligo–C as a real sensor, a preliminary test strip was carried out. Common filter paper was absorbed with the Zn–Oligo–C solution (50 μL, 1 mM in DMSO). After the exposure to DMMP vapors (40 ppm, in a closed vial at room temperature), a clear decrease of color intensity was observed (Figure 10). This preliminary result paves the way for the employment of this new receptor as a sensor prototype.

## 3. Materials and Methods

*General experimental methods*. The NMR experiments were carried out at 27 °C on a Varian UNITY Inova 500 MHz spectrometer (^1^H at 499.88 MHz, Varian-Agilent, Santa Clara, CA, USA) equipped with pulse field gradient module (Z axis) and a tunable 5 mm Varian inverse detection probe (ID-PFG). A JASCO V-560 UV-Vis spectrophotometer (Mettler Toledo, Novate Milanese, Italy) equipped with a 1 cm path-length cell was used for the UV-Vis measurements. Luminescence measurements were carried out using a Cary Eclipse Fluorescence spectrophotometer (Agilent, Santa Clara, CA, USA) with resolution of 0.5 nm, at room temperature. The emission was recorded at 90° with respect to the exciting line beam using 10:5 slit-widths for all measurements. The fluorescence quantum yields were calculated by using*N*-butyl-4-butylamino-1,8-naphthalimide as standard. All chemicals were reagent grade and used without further purification. ^13^C NMR characterizations of oligomers were precluded due to the scarce solubility of the compounds.

*Gel permeation chromatography*. A PL-GPC 110 (Polymer Laboratories) thermostat system, equipped with three PL-gel 5 mm columns (two Mixed-D and one Mixed-E) attached in series, was used. The analyses were performed at 35 ± 0.1 °C using THF as eluent at a flow rate of 1 mL/min. A differential refractometer (Polymer Laboratories, Wyatt Technology, Dernbach, Germany) was used as detector.

*Procedure for epsilon calculation*. UV-Vis spectra of four solutions of the hosts at different concentration, from 1 × 10^−5^ M to 4 × 10^−5^ M, were measured at 25 °C. Data treatment allowed for the calculation of epsilon for each absorption band.

*Procedure for fluorescence titrations.* Two stock solutions of host and guest (1.0 × 10^−3^ M) in dry solvent were prepared. From these, progressive amounts of guest solution were added to the host (from 0 to 6 eq.), and emission spectra were recorded at 25 °C. Fluorescence titration with Zn–Oligo–A, –B, and –C were carried out using λ_ex_ = 365 nm in dry DMSO and recorded at λ_em_= 480 nm at 25 °C. With this data treatment, the apparent binding affinities of receptors with DMMP were estimated using the HypSpec (version 1.1.33, Protonic Software, Florence, Italy) [45,46], a software designed to extract equilibrium constants from potentiometric and/or spectrophotometric titration data. HypSpec starts with an assumed complex formation scheme and uses a least-squares approach to derive the spectra of the complexes and the stability constants. χ^2^ test (chi-square) was applied, where the residuals follow a normal distribution (for a distribution approximately normal, the χ^2^ test value is around 12 or less). In all of the cases, values of χ^2^ ≤ 10 were found, as obtained by 3 independent measurements sets.

*DOSY experiments*. Diffusion Ordered SpectroscopY (DOSY) NMR was used to determine the presence of monomeric or higher species in solution. The DOSY technique provides information about the size of the molecular aggregate in solution. Furthermore, the diffusion coefficient value could be associated to the molecular weight by the mathematic treatment recently described [42,43]. DOSY experiments on Oligo–Salen–A in DMSO-*d*_6_ (10 mM) showed a diffusion coefficient of 3.41 × 10^−10^ m^2^ s^−1^, corresponding to a calculated molecular weight of ca. 4650 (prevalence of decameric form, the experimental molecular weight of dimer is 4330). While in DMSO-*d*_6_, a solution of Oligo–Salen–B (10 mM) showed a diffusion coefficient of 4.23 × 10^−10^ m^2^ s^−1^, (calculated molecular weight of 2760, prevalence of hexameric form, the experimental molecular weight is 2598). Oligo–Salen–C showed a diffusion coefficient of 5.29 × 10^−10^ m^2^ s^−1^ (calculated molecular weight of 1640, prevalence of tetrameric form, the experimental molecular weight is 1732), relative to a solution of ligand in DMSO–*d*_6_ (10 mM).

*Synthesis of Oligo–Salen–A*: 5,5’–methylenebis–2–hydroxybenzaldehyde (0.150 g, 0.58 mmol) [39], 2–hydroxy–benzhaldehyde (31 μL, 0.29 mmol), and *meso*–1,2–diphenylethylenediamine (0.160 g, 0.73 mmol) were stirred in anhydrous toluene (22 mL) at room temperature for 48 h. Then the solvent was removed under reduced pressure, thus leading to the Oligo-Salen-A (0.380 g) as pale yellow crystals, then washed with hexane: ^1^H NMR (500MHz, CDCl_3_) δ 13.1–12.8 (bs, 2H, OH), 8.1–7.9 (m, 2H, CH=N), 7.4–6.6 (m, 18H, ArH), 4.8–4.6 (bs, 2H, Ar-CH-N), 3.7 (bs, 2H, Ar-CH_2_-Ar) ppm. Anal. Calcd. for C_290_H_260_N_20_O_20_: C, 80.16; H, 6.03; N, 6.45. Found C, 80.12; H, 6.01; N, 6.41.

*Synthesis of Oligo–Salen–B:* 5,5’–methylenebis–2–hydroxybenzaldehyde (0.150 g, 0.58 mmol), 2–hydroxy–benzhaldehyde (62 μL, 0.58 mmol), and *meso*–1,2–diphenylethylenediamine (0.192 g, 0.87 mmol) were stirred in anhydrous toluene (22 mL) at room temperature for 72 h. Then the solvent was removed under reduced pressure, thus leading to the Oligo–Salen–B (0.230 g) as pale yellow crystals, then washed with hexane: ^1^H NMR (500MHz, CDCl_3_) δ 13.1–12.8 (bs, 2H, OH), 8.1–7.9 (m, 2H, CH=N), 7.4–6.7 (m, 18H, ArH), 4.8–4.6 (bs, 2H, Ar-CH-N), 3.7 (bs, 2H, Ar-CH_2_-Ar) ppm. Anal. Calcd. for C_174_H_156_N_12_O_12_: Found C, 80.14; H, 6.00; N, 6.39.

*Synthesis of Oligo–Salen–C:* 5,5’–methylenebis–2–hydroxybenzaldehyde (39 mg, 0.15 mmol), 2–hydroxy–benzhaldehyde (32 μL, 0.30 mmol), and *meso*–1,2–diphenylethylenediamine (66 mg, 0.30 mmol) were stirred in anhydrous toluene (22 mL) at room temperature for 72 h. Then the solvent was removed under reduced pressure, thus leading to the Oligo–Salen–C (40 mg) as yellow crystals, then washed with hexane: ^1^H NMR (500MHz, CDCl_3_) δ 13.1–12.8 (bs, 2H, OH), 8.1–7.9 (m, 2H, CH=N), 7.4–6.7 (m, 18H, ArH), 4.8–4.6 (bs, 2H, Ar-CH-N), 3.7 (bs, 2H, Ar-CH_2_-Ar) ppm. Anal. Calcd. for C_116_H_104_N_8_O_8_: Found C, 80.11; H, 6.02; N, 6.44.

*Synthesis of Zn–Oligo–A:* To a solution of the Oligo–Salen–A ligand 0.315 g (0.73 mmol) in dry tetrahydrofuran (15 mL), 900 μL (0.90 mmol) of Et_2_Zn 1 M in hexane was added. The reaction mixture was stirred for 48 h at room temperature, thus affording Zn–Oligo–A (0.350 g) as a brown-yellow precipitate, which was collected by filtration. ^1^H NMR (500MHz, CDCl_3_) δ 8.2–8.0 (m, 2H, H-C=N), 7.2–6.3 (m, 17H, Ar-H), 5.1–5.0 (m, 2H, Ar-CH-N), 3.5 (bs, 2H, Ar-CH_2_-Ar) ppm. Anal. Calcd. for C_290_H_240_N_20_O_20_Zn_10_: C, 69.96; H, 4.86; N, 5.63. Found C, 69.92; H, 4.81; N, 5.60.

*Synthesis of Zn–Oligo–B:* To a solution of Oligo–Salen–B ligand 0.200 g (0.46 mmol) in dry tetrahydrofuran (19 mL), 680 μL (0.60 mmol) of Et_2_Zn 1 M in hexane was added. The reaction mixture was stirred for 48 h at room temperature, thus affording Zn–Oligo–B (0.190 g) as a brown-yellow precipitate, which was collected by filtration. ^1^H NMR (500MHz, CDCl_3_) δ 8.2–8.0 (m, 2H, H-C=N), 7.3–6.3 (m, 17H, Ar-H), 5.1–4.9 (m, 2H, Ar-CH-N), 3.5 (bs, 2H, Ar-CH_2_-Ar) ppm. Anal. Calcd. for C_174_H_144_N_12_O_12_Zn_6_: Found C, 69.93; H, 4.85; N, 5.59.

*Synthesis of Zn–Oligo–C:* Oligo–Salen–C ligand (38 mg, 0.080 mmol) was dissolved in dry tetrahydrofuran (6 mL). To this solution, 100 μL (0.10 mmol) of Et_2_Zn 1 M in hexane was added. The reaction mixture was stirred for 24 h at room temperature, thus affording Zn–Oligo–C (23 mg) as a yellow precipitate, which was collected by filtration. ^1^H NMR (500MHz, CDCl_3_) δ 8.2–8.0 (m, 2H, H-C=N), 7.3–6.3 (m, 17H, Ar-H), 5.1–5.0 (m, 2H, Ar-CH-N), 3.5 (bs, 2H, Ar-CH_2_-Ar) ppm. Anal. Calcd. for C_116_H_96_N_8_O_8_Zn_4_: Found C, 69.91; H, 4.80; N, 5.55.

## 4. Conclusions

In this manuscript we describe the synthesis and characterization of three new Zn–oligomer–based Salen complexes. The easy synthetic protocols based on the mixing of reagents with a proper stoichiometric ratio allowed for the obtainment of oligomers with four, six, and ten Salen units. These oligomers were tested as receptors for DMMP and we found excellent results with the shorter oligomer. In fact, the shorter oligomer showed the highest binding constant value with respect to the other sensors used for the non-covalent detection of DMMP. These results suggest the possibility to employ the oligomer hosts in the CWA detection, in order to obtain final solid devices with higher affinity for the analytes.

## Figures and Tables

**Table 1:** DOSY results.

Oligomer ^a^	D (× 10^−10^ m^2^s^−1^)	Calcd. Mn ^b^	Degree of Polymerization ^c^
Oligo–Salen–A	3.41	4650	~10
Oligo–Salen–B	4.23	2760	~6
Oligo–Salen–C	5.29	1640	~4

^a^ Concentration of sample = 1 × 10^−3^ M in CDCl_3_; ^b^ estimated by using the method described in Ref. [41]; ^c^ Degree of polymerization = Mn/MM_R.U._

**Table 2:** Binding constant values of oligomeric and monomeric receptors, towards DMMP in DMSO solution.

Guest: DMMP	Zn–Oligo–A	Zn–Oligo–B	Zn–Oligo–C	Zn–5–*t*but (Monomer)
**LogK ^a^**	4.86	4.98	5.69	4.33 [39]

^a^ calculated using HypSpec 1.1.33.

## Supplementary Materials

The following are available online at https://www.mdpi.com/1420-3049/24/11/2160/s1: NMR, UV-Vis, emission spectra, fluorescence titration.
