# Supplementary material for: Supramolecular Detection of a Nerve Agent Simulant by Fluorescent Zn–Salen Oligomer Receptors
Source: Molecules. 2019 Jun 8;24(11):2160. doi: 10.3390/molecules24112160 (PMC6600340; doi:10.3390/molecules24112160)
Supplement: Supplementary file 1 [file molecules-24-02160-s001.pdf]

Supramolecular Detection of a Nerve Agent Simulant by Fluorescent Zn–Salen Oligomer Receptors

Roberta Puglisi *, Placido G. Mineo, Andrea Pappalardo, Antonino Gulino and Giuseppe Trusso Sfrazzetto*

Table of content

^{1}H NMR spectra	S2-S4
UV-Vis and Fluorescence spectra	S5-S6
Fluorescence titration	S7
Hyp Spec output data	S7-S8
Selectivity	S9
MALDI-TOF spectrum	S9

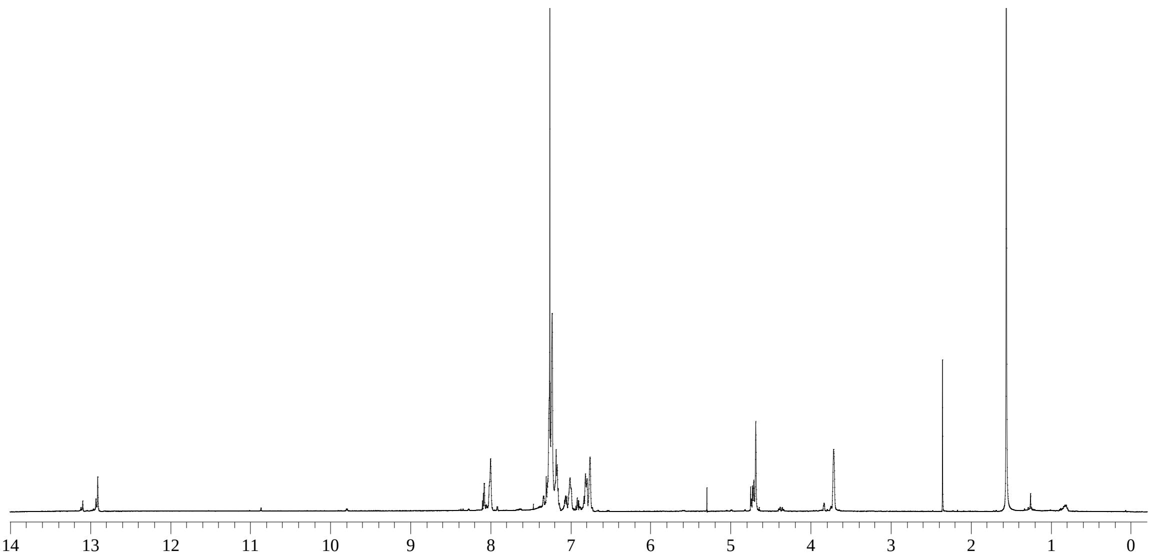

Figure S1. ^{1}H NMR spectrum of Oligo–Salen–A in $CDCl_3$

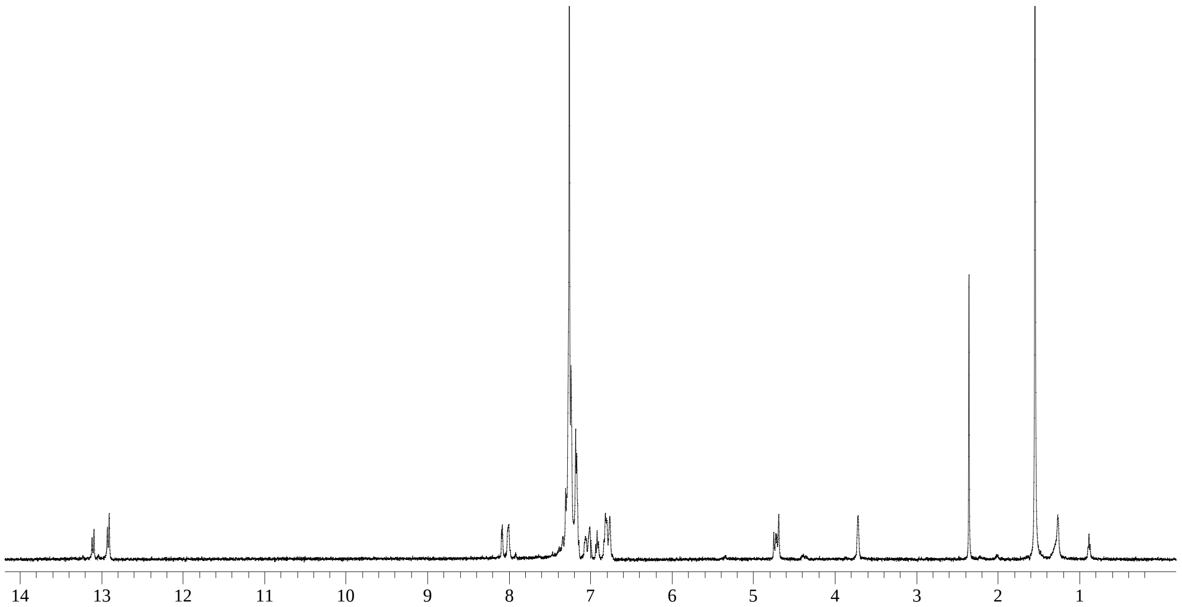

Figure S2. ^{1}H NMR spectrum of Oligo–Salen–B in $CDCl_3$

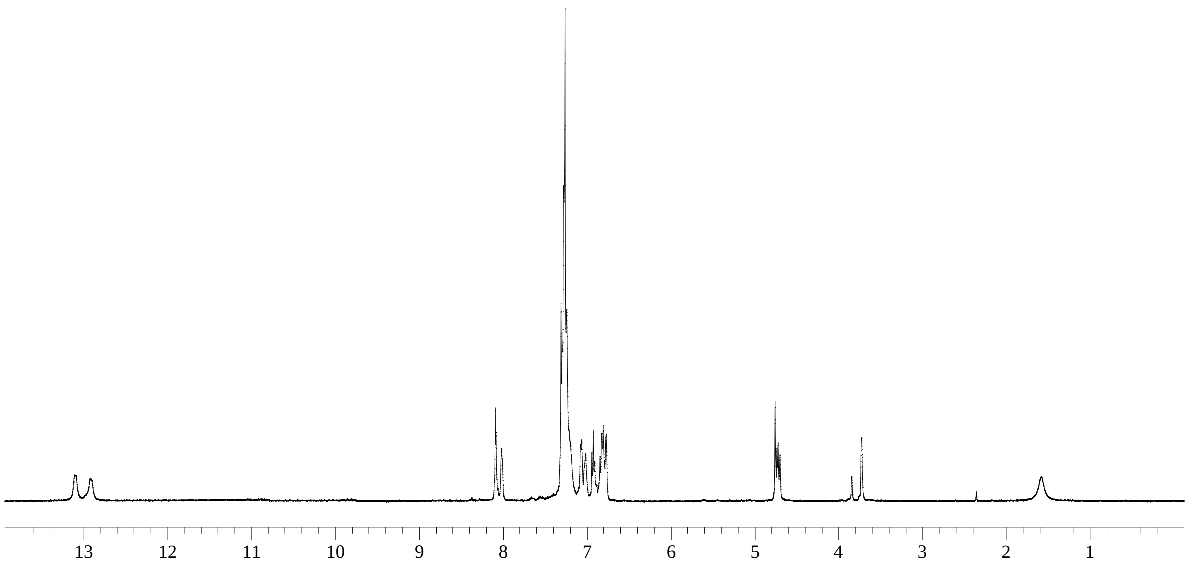

Figure S3. ^{1}H NMR spectrum of Oligo–Salen–C in $CDCl_3$

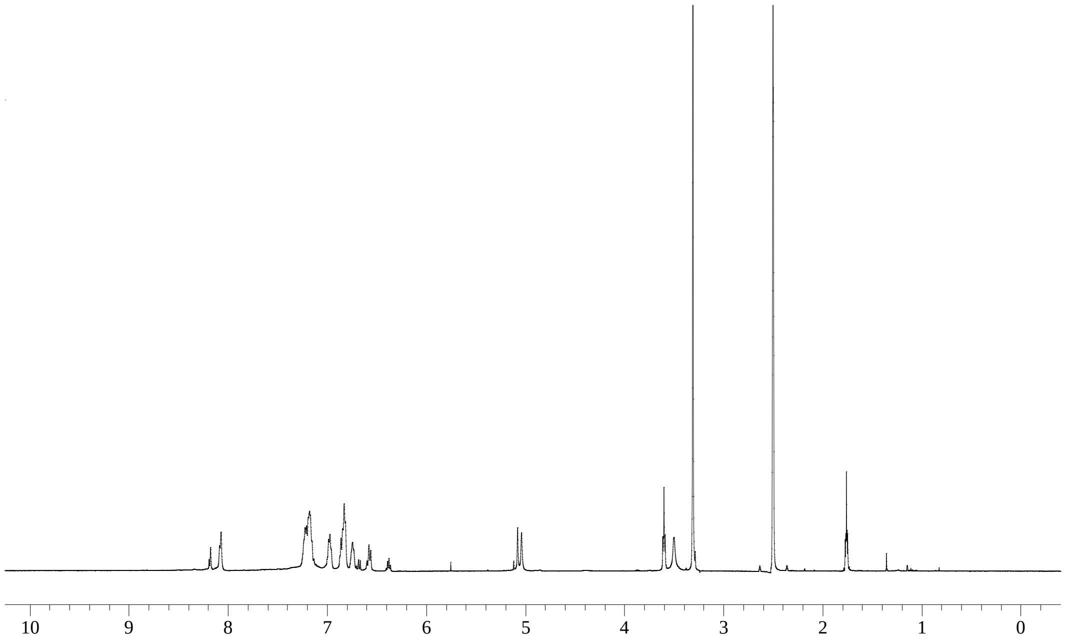

Figure S4. ^{1}H NMR spectrum of Zn–Oligo–A in DMSO-d_6

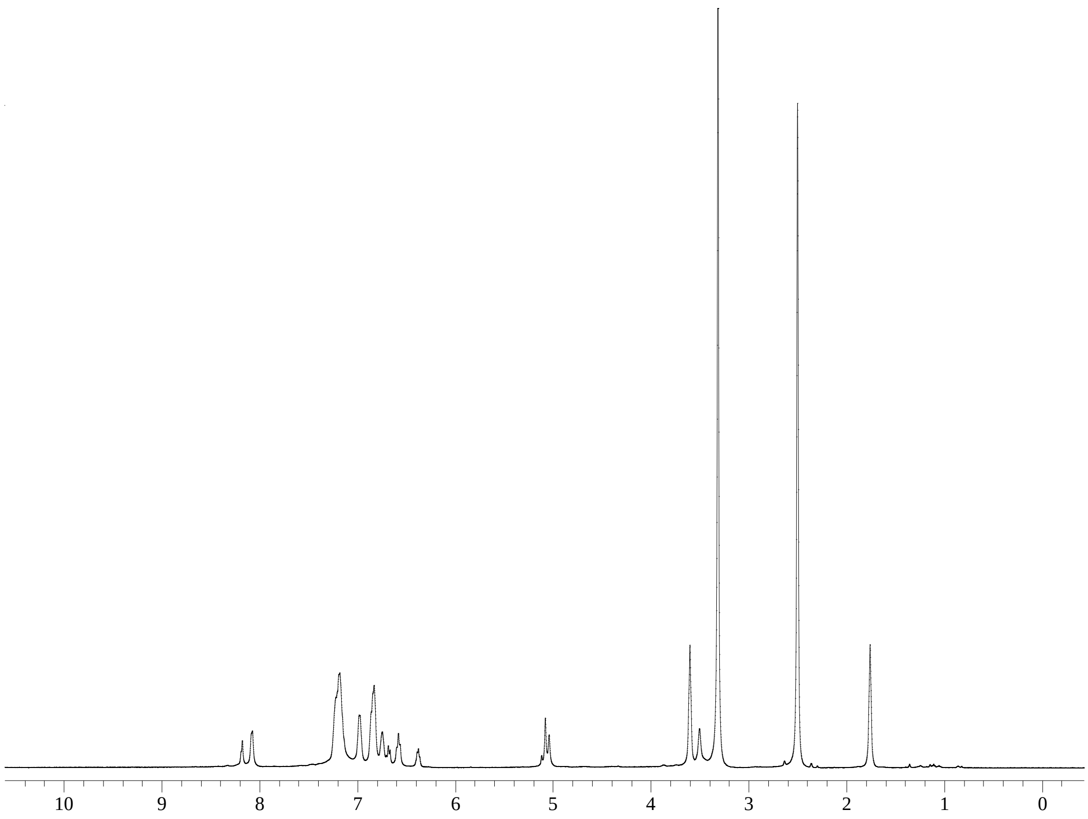

Figure S5. ^{1}H NMR spectrum of Zn–Oligo–B in DMSO-d_6

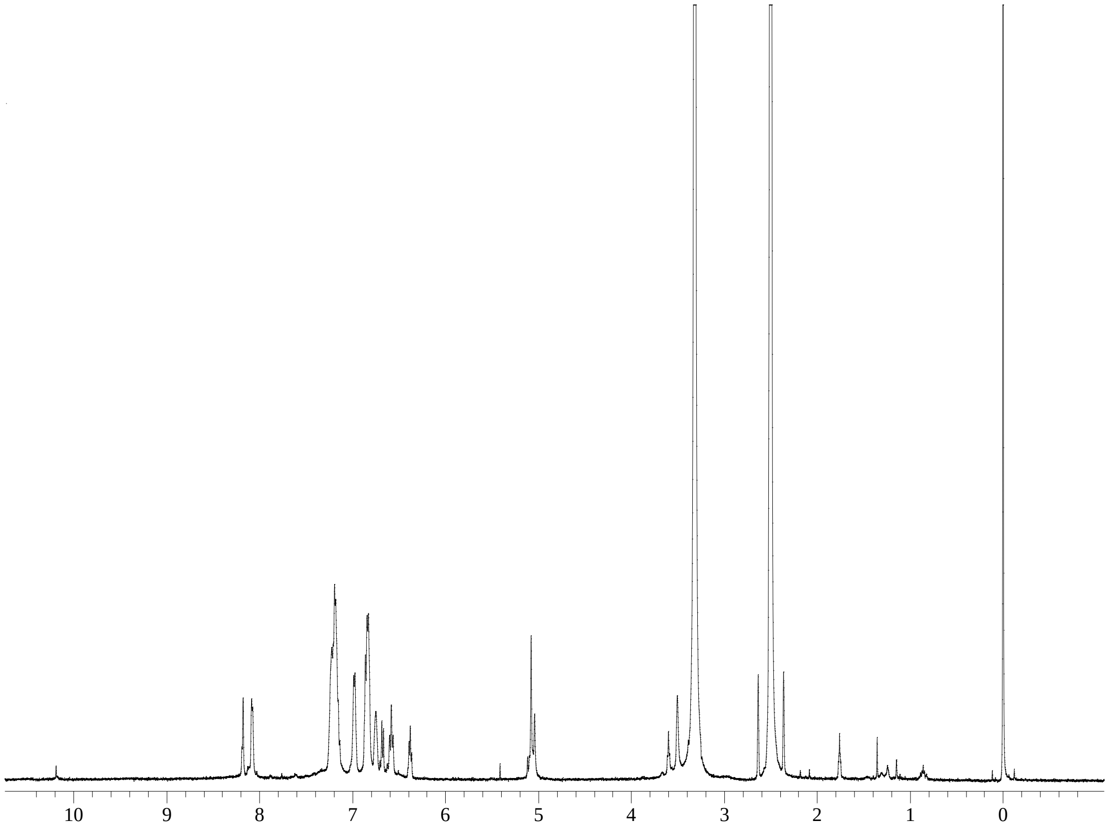

Figure S6. ^{1}H NMR spectrum of Zn–Oligo–C in DMSO-d_6

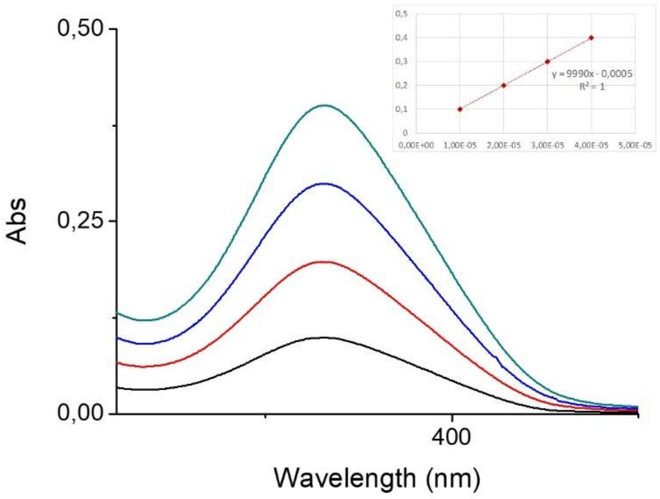

Figure S7. UV-Vis spectra of Zn–Oligo–A in DMSO (1 x 10^{-5} M – 4 x 10^{-5} M). Inset shows ε calculation.

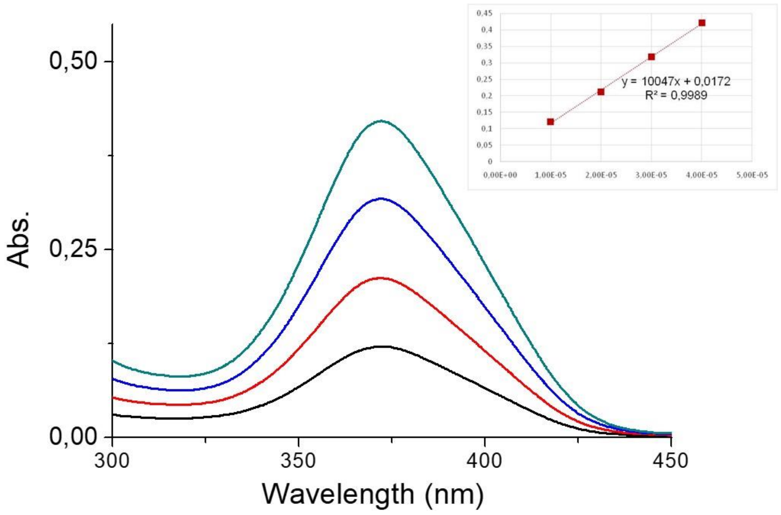

Figure S8. UV-Vis spectra of Zn–Oligo–B in DMSO (1 x 10^{-5} M – 4 x 10^{-5} M). Inset shows ε calculation.

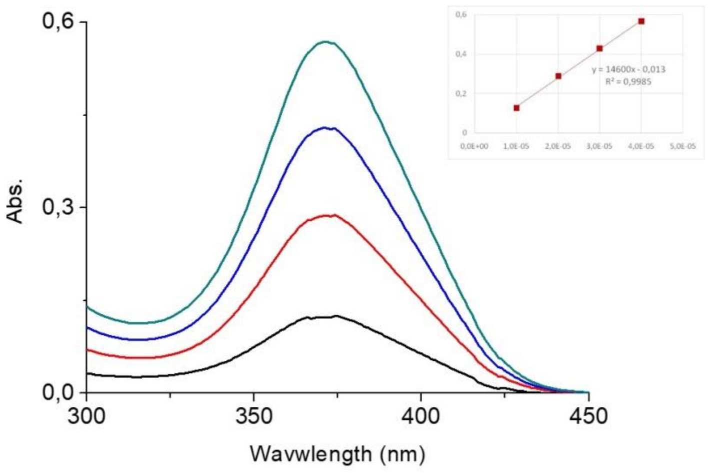

Figure S9. UV-Vis spectra of Zn–Oligo–C in DMSO (1 x 10^{-5} M – 4 x 10^{-5} M). Inset shows ε calculation.

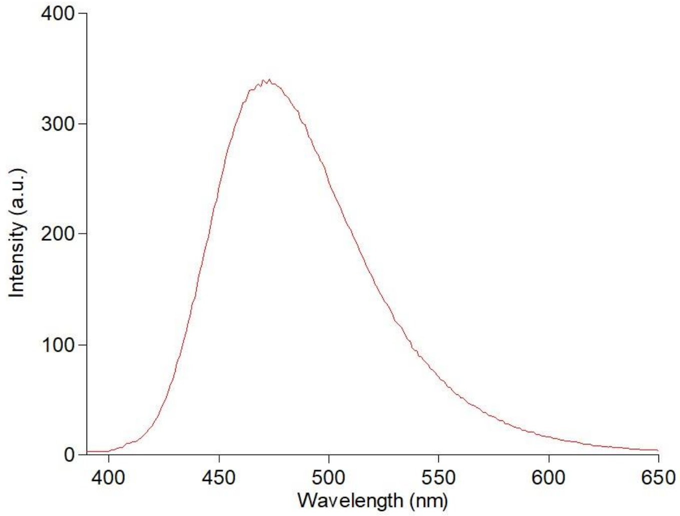

Figure S10. Fluorescence spectra of Zn–Oligo–A in DMSO (1 x 10^{-5} M)

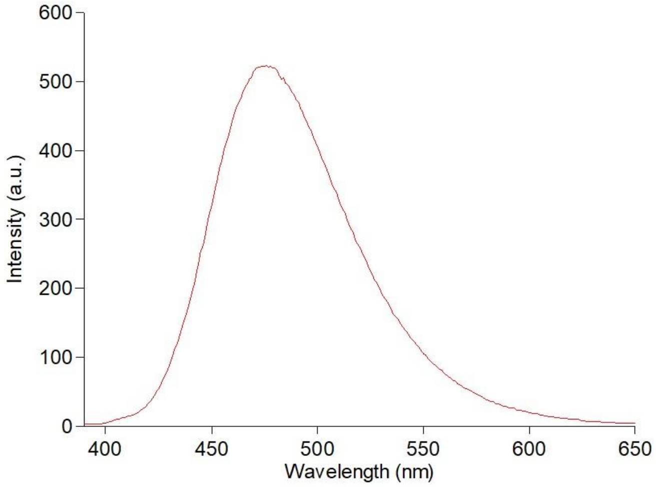

Figure S11. Fluorescence spectra of Zn–Oligo–B in DMSO (1 x 10^{-5} M)

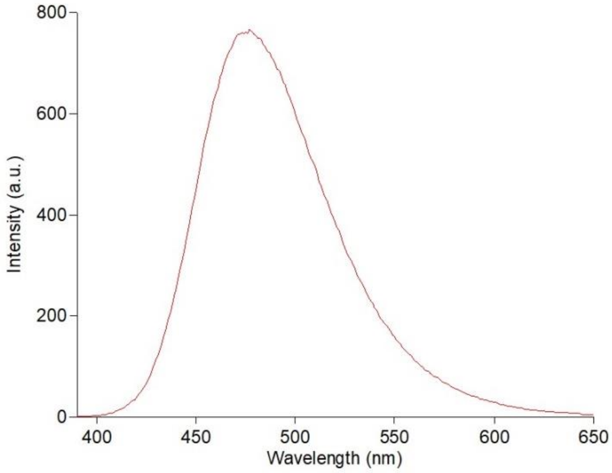

Figure S12. Fluorescence spectra of Zn–Oligo–C in DMSO (1 x 10^{-5} M)

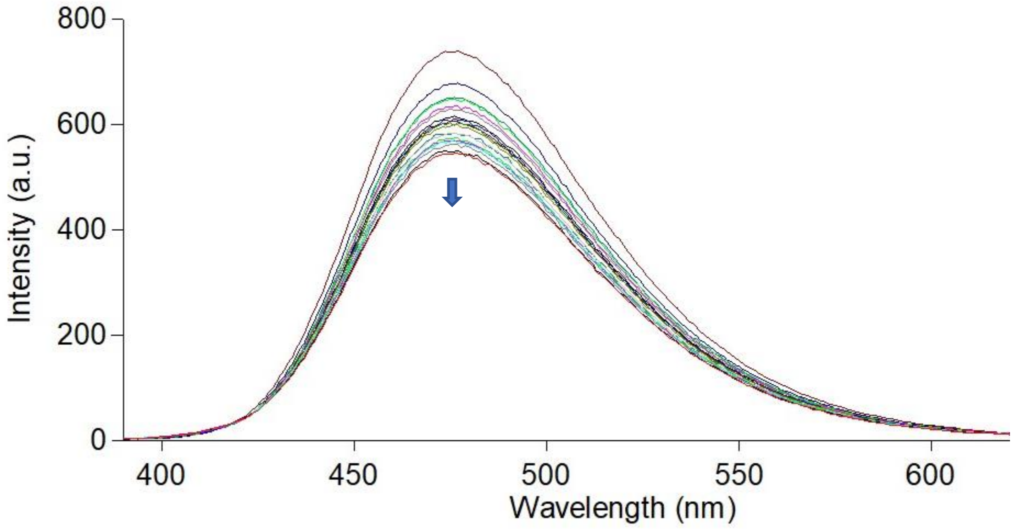

Figure S13. Representative fluorescence titration of Zn–Oligo–C vs DMMP in DMSO ([Host] = 1 x 10^{-5} M, [Guest] = from 1 to 6 eq.)

BINDING CONSTANT CALCULATION

Host: Zn–Oligo–A; Guest DMMP

```
HypSpec output file:

Converged in 1 iterations with sigma = 0,030713

                      standard
Log beta    value     deviation
AB          4.8567    0.0698
```

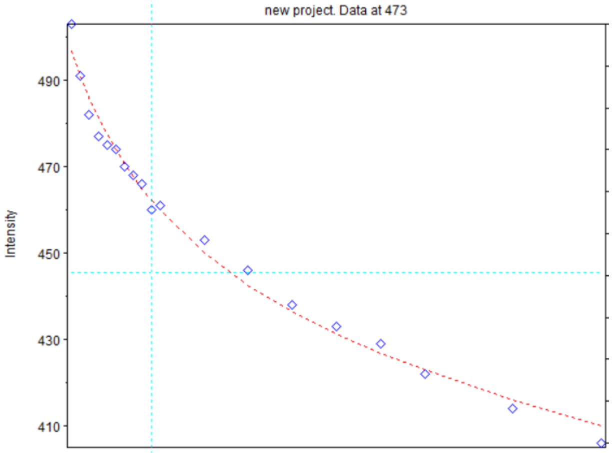

Figure S14.

Host: Zn–Oligo–B; Guest: DMMP

```
HypSpec output file:

Converged in 1 iterations with sigma = 0,030713

                      standard
Log beta    value     deviation
AB          4.9756    0.098
```

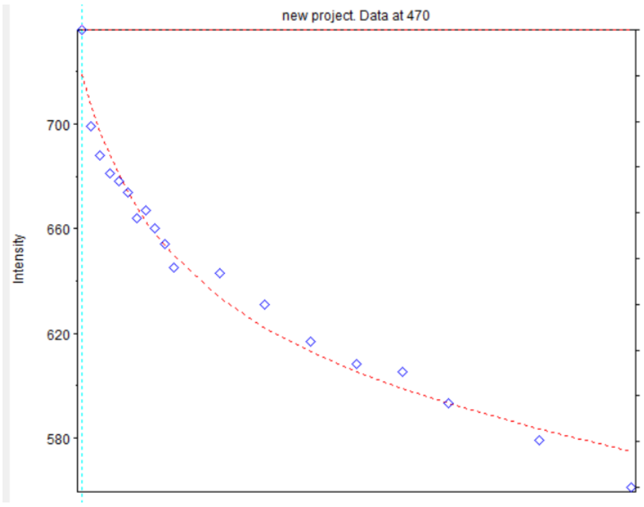

Figure S15.

Host: Zn–Oligo–C; Guest: DMMP

```
HypSpec output file:

Converged in 1 iterations with sigma = 0,030713

                      standard
Log beta    value     deviation
AB          5.6891    0.0409
```

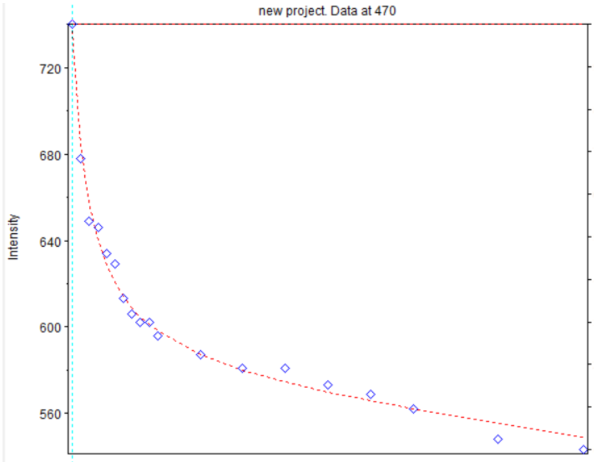

Figure S16.

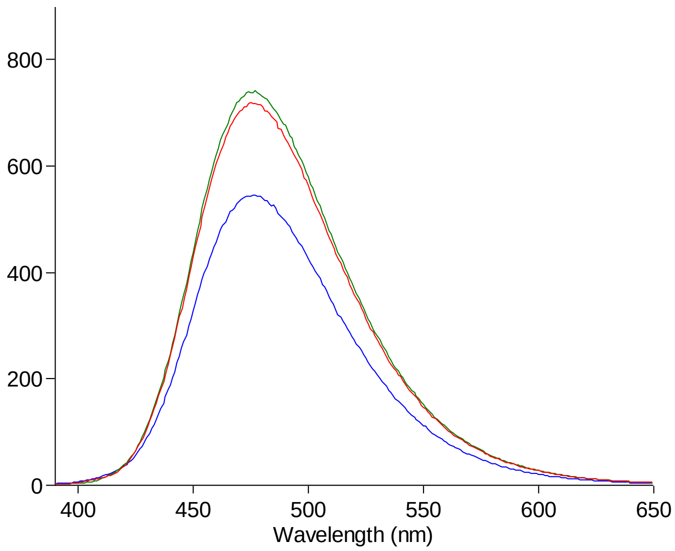

Figure S17. Selectivity tests. Emission spectra of: *i*) Zn–Oligo–C (red line, 1 x 10^{-5} M in DMSO), *ii*) Zn–Oligo–C after 10 minutes of air bubbling (green line), *iii*) Zn–Oligo–C after 10 minutes of air bubbling and 6 eq. of DMMP (blue line).

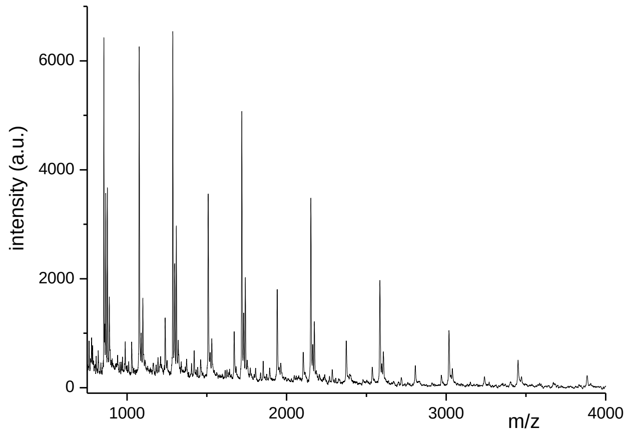

Figure S18: MALTI-TOF spectrum of Oligo–Salen–C.
